# Optimal designs for population pharmacokinetic studies of the partner drugs co-administered with artemisinin derivatives in patients with uncomplicated falciparum malaria

**DOI:** 10.1186/1475-2875-11-143

**Published:** 2012-07-11

**Authors:** Kris M Jamsen, Stephen B Duffull, Joel Tarning, Niklas Lindegardh, Nicholas J White, Julie A Simpson

**Affiliations:** 1 Centre for Molecular, Environmental, Genetic and Analytic Epidemiology, School of Population Health, The University of Melbourne, Melbourne, Australia; 2 School of Pharmacy, The University of Otago, Dunedin, New Zealand; 3 Mahidol-Oxford Tropical Medicine Research Unit, Mahidol University, Bangkok, Thailand; 4 Centre for Tropical Medicine, Oxford, UK

**Keywords:** Artemisinin-based combination therapy, Partner drugs, Optimal design

## Abstract

**Background:**

Artemisinin-based combination therapy (ACT) is currently recommended as first-line treatment for uncomplicated malaria, but of concern, it has been observed that the effectiveness of the main artemisinin derivative, artesunate, has been diminished due to parasite resistance. This reduction in effect highlights the importance of the partner drugs in ACT and provides motivation to gain more knowledge of their pharmacokinetic (PK) properties via population PK studies. Optimal design methodology has been developed for population PK studies, which analytically determines a sampling schedule that is clinically feasible and yields precise estimation of model parameters. In this work, optimal design methodology was used to determine sampling designs for typical future population PK studies of the partner drugs (mefloquine, lumefantrine, piperaquine and amodiaquine) co-administered with artemisinin derivatives.

**Methods:**

The optimal designs were determined using freely available software and were based on structural PK models from the literature and the key specifications of 100 patients with five samples per patient, with one sample taken on the seventh day of treatment. The derived optimal designs were then evaluated via a simulation-estimation procedure.

**Results:**

For all partner drugs, designs consisting of two sampling schedules (50 patients per schedule) with five samples per patient resulted in acceptable precision of the model parameter estimates.

**Conclusions:**

The sampling schedules proposed in this paper should be considered in future population pharmacokinetic studies where intensive sampling over many days or weeks of follow-up is not possible due to either ethical, logistic or economical reasons.

## Background

Despite substantial progress in the last decade, malaria remains a major global health problem [1]. In 2009 there were an estimated 225 million cases and 781,000 malaria-related deaths [2]. Artemisinin-based combination therapy (ACT) is recommended as first-line treatment for uncomplicated *falciparum* malaria [3]. The primary antiparasitic agent in ACT is the artemisinin derivative, which is quickly absorbed by and eliminated from the body, and destroys the majority of detectable parasites within the first 3 days of treatment. The partner drug(s) co-administered with the artemisinin derivative is less effective but slowly eliminated, and thus provides residual antiparasitic activity in the patient. Additionally, the partner drug offers protection to the artemsinin derivative from rare mutant parasites that have developed resistance [3].

Though the artemisinin derivitives are generally effective, it has been observed in Cambodia that the effectiveness of the main artemisinin derivative, artesunate, has been diminished due to parasite resistance [4]. These findings highlight the necessity and importance of the partner drugs in ACT, and provide impetus for gaining more knowledge of their pharmacokinetic (PK) properties. This knowledge can ultimately be used to optimize dosing regimens, particuarly for high risk groups such as pregnant women and children.

The PK properties of a drug can be characterized for intended target populations via population PK studies. For anti-malarial drugs, these studies need to be large enough and designed in order to be representative for the target population(s) so that the expected concentration-time profile and between-subject variability parameters can be characterized adequately [5]. From a modelling perspective, it would be ideal to propose study designs with intensive blood sampling, but often this is not realistic for patients presenting with *falciparum* malaria. Intensive sampling over the entire follow-up period is logistically difficult, especially in rural settings where outpatients attend few follow-up visits [5]. However, population PK modelling allows information to be “borrowed” across individuals to obtain parameter estimates [6,7]; therefore it is reasonable to propose study designs with fewer samples per patient but a sufficiently large number of patients. The key aspect of such a design lies with the timing of blood samples, since the times at which the samples are taken must provide sufficient information for precise estimation of model parameters.

Optimal design methodology has been developed for designing population PK studies [8]; the method analytically determines a blood sampling schedule that provides precise estimates of the model parameters. Importantly, the methodology allows economical use of resources [6], for which in the case of the partner drugs is fewer samples per patient but with more patients enrolled. The overall aim of this work was to determine optimal blood sampling schedules that can be used to study the partner drugs of the most widely used ACT that are economical, efficient, and appropriate for all target populations.

## Methods

### Determination of the optimal designs

For each partner drug (mefloquine, lumefantrine, piperaquine and amodiaquine), an optimal design for a combined study population of non-pregnant adults, pregnant women and children was determined using D-optimal design theory. In brief, a D-optimal design is the design that maximizes the determinant of the population Fisher Information matrix, yielding the smallest possible standard errors. For full details, see [8].

The designs were determined using POPT [9], which implements D-optimal design methodology. Thus for each partner drug:

1. population PK models (and the associated parameter values) for non-pregnant adults, pregnant women and children were identified from the literature and entered into POPT,

2. the key constraints of 100 patients, five plasma samples per patient (including one sample on the seventh day of treatment) and two sampling designs (groups) were specified,

3. optimal sampling times for the combined population of pregnant women, non-pregnant adults and children were determined, and

4. sampling windows (time intervals containing the optimal sampling times) were derived.

The key specifications of 100 patients and five blood samples per patient were consistent with the sampling constraints reported in a survey administered to 22 malaria researchers with extensive experience in conducting PK studies in Asia and Africa [10] and World Health Organization guidelines for sampling schemes for pharmacokinetic studies of anti-malarial drugs [5]. A fixed sample on the seventh day of treatment was chosen since it has been recommended that a day 7 plasma drug concentration should be collected as a routine part of anti-malarial drug trials, particularly for the partner drugs given with artemisinin derivatives [5,11]. The day 7 concentration is generally within the assay limits of quantification and has been shown to be predictive of treatment failure [5,11]. Two sampling designs (i.e. two groups with different sampling schedules; n = 50 for each group) were specified to provide (i) adequate coverage of the PK profiles, since each patient contributes only five samples and the drugs are eliminated over several weeks, and (ii) flexibility with sampling, since one sampling schedule may be too restrictive for an outpatient cohort. The dosing regimens for all drugs were in concordance with the World Health Organization guidelines for the treatment of malaria [3].

**Figure 1:**
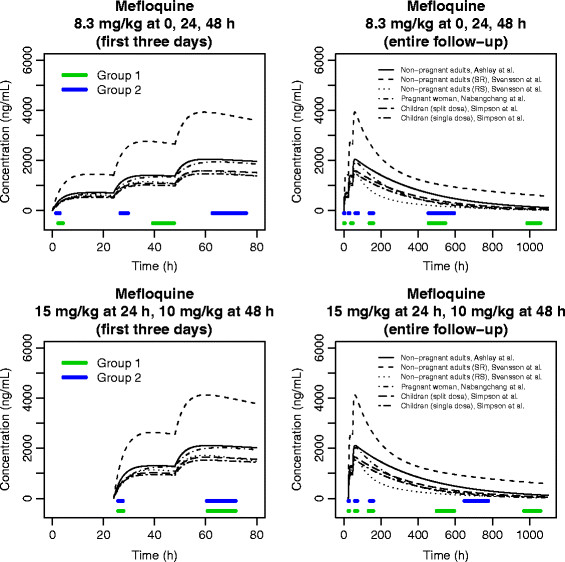
**Structural pharmacokinetic models considered for the mefloquine optimal designs, with parameter values set to the reported population estimates.** The optimal sampling windows are displayed on the time axes.

**Figure 2:**
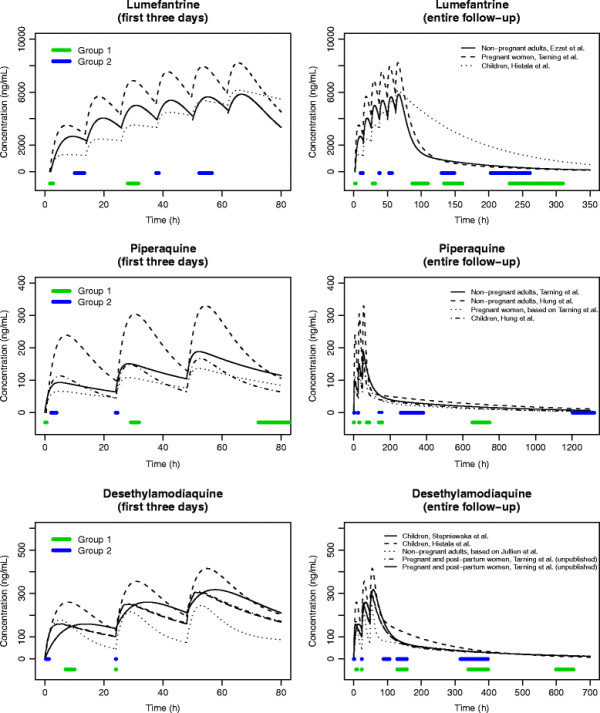
**Structural pharmacokinetic models considered for the lumefantrine, piperaquine and desethylamodiaquine optimal designs, with parameter values set to the reported population estimates.** The optimal sampling windows are displayed on the time axes.

The structural PK models that were considered for each design are displayed in Figures 1 and 2 (in the figures, the PK parameters were set to the reported population values). The mefloquine design was based on one-compartment population PK models reported for Thai children [12] and pregnant women [13], as well as two-compartment models reported for African adults [14]. Two optimal designs were determined for mefloquine: one for the dosing regimen of 8.3 mg/kg at 0, 24 and 48 h and another for 15 mg/kg at 24 h and 10 mg/kg at 48 h. The optimal design for lumefantrine was based on two-compartment models reported for Thai adults [15] and pregnant women [16], and a one-compartment model reported for Tanzanian children [17]. The design for piperaquine was based on two-compartment models reported for adults and children in Cambodia [18] and Thailand [19]. At the time the designs were determined there were no published population PK models for piperaquine in pregnant women, so PK profiles for this group were derived from results reported in [19] and a pseudo-pregnant state was simulated by increasing the clearance and volume parameters by 40 and 30%, respectively. For amodiaquine, the design was determined for the active metabolite, desethylamodiaquine. The design was based on two-compartment models reported in African children [20,21] and adults [22], as well as an unpublished two-compartment model for Thai pregnant and post-partum women (listed in Additional file 1 with a full description of how this model was entered into POPT). The model reported in [22] was approximated by a two-compartment model based on an analysis of simulated desethylamodiaquine concentrations from the reported parent-metabolite model. For full details of each model see [12-23].

For each structural model entered into POPT, the PK parameter values were set to the population mean estimates (displayed graphically in Figures 1 and 2) and the lower and upper bounds of their respective 95% confidence intervals. A weighting of 95% was allocated to the models where the PK parameters were set to the population estimates and likely ranges of age and/or body weight were considered for each model. The between-subject variability (BSV) parameters were set to the reported population mean estimates, and if not reported the BSV of the absorption rate constant (*k*_*a*_) was set to 50% and the BSVs for other parameters were set to 30%. These values were based on a summary of the literature of PK studies for anti-malarial drugs [5] and the authors’ previous experience with other drugs. The proportional and additive residual variability parameters were also set to their reported population estimates. If the additive component was not reported it was set to a plausible value from another study of the same drug, and if the proportional component was not reported it was set to 10% (again based on [5] and previous experience with other drugs).

To ensure the designs were clinically feasible, sampling windows were derived in POPT. A sampling window is a time interval of acceptable sub-optimality such that any set of samples taken within the windows ensures minimal impact on the standard errors of the estimated parameters. For all designs, the windows were constructed such that there would be at worst a 20% relative increase in the standard errors compared to the optimal sampling times.

### Evaluation of the optimal designs

The robustness of the optimal designs was evaluated by a simulation-estimation procedure that was automated in NONMEM 7.1 [24]. So for each optimal design:

1. 100 datasets were simulated, where each dataset consisted of 100 virtual patients: 33 non-pregnant adults, 33 pregnant women and 34 children. This was done to mimic the clinically realistic scenario of adults and children being enrolled in the same study. Body weights for each of these groups were simulated from distributions that were consistent with those reported in the literature.

2. Individual drug concentrations were simulated at the optimal sampling times from a respective population PK model.

3. Each simulated dataset was analysed with NONMEM using the FOCE with INTERACTION method. The M3 method was used when 10% or more of the simulated concentrations fell below the lower limit of quantification [24,25]. For each dataset, the non-pregnant adults, pregnant women and children were analysed separately. This was done to mimic a “worst case” scenario where the patient groups could not be modelled together (e.g. with indicator variables on the PK parameters). An estimation run was considered successful if NONMEM declared successful minimization of the objective function or reported a termination due to rounding errors (error code 134). The latter condition was considered a “successful” run since it can usually be remedied by altering the starting values.

4. For each estimated parameter, empirical percent relative standard errors (%RSEs) were computed. This was done by dividing the standard deviation of the estimates from the analyses by the median and multiplying by 100. The empirical %RSEs were compared with the expected %RSEs from POPT.

For the mefloquine design, data were simulated from and analysed with one-compartment models reported in [12,13,23] (children, non-pregnant adults and pregnant women). Additionally, non-pregnant adult data were simulated from and analysed with a two-compartment model reported in [14] (SR enantiomer). For the lumefantrine design PK data for non-pregnant adults and pregnant women were simulated from and analysed with two-compartment models reported in [15] and [16], respectively. Lumefantrine profiles for children were simulated from and analysed with the one-compartment model reported in [17]. For the piperaquine design, non-pregnant adult and paediatric data were simulated from and analysed with two-comparment models reported in [19] and [18] (respectively). In addition, piperaquine profiles for children, pregnant and non-pregnant women were simulated from very recent models reported in [26] and [27] (three-compartment models with n-transit absorption) and analysed with two-compartment models. For the desethylamodiaquine design, data for children were simulated from and analysed with the two-compartment model reported in [21]. For the adults, desethylamodiaquine concentrations were simulated from and analysed with the unpublished two-compartment model provided by JT. This model assumed that pregnancy did not affect the PK of desethylamodiaquine, therefore only a single adult simulation-estimation procedure was performed (n = 66, with allometric scaling on clearance and volume parameters). See Additional file 1 for more details on how this model was employed for the evaluation of this design. The simulation-estimation procedure was not performed for the model approximated from [22] since this model was based on simulated concentrations.

## Results

### Optimal designs

Table 1 shows the optimal sampling times and sampling windows for each partner drug design and Figures 1 and 2 display the sampling windows graphically. For the mefloquine, piperaquine and desethylamodiaquine designs, it was specified in POPT for all patients to have one sample taken on the first and second days of treatment and three samples taken over the remainder of the follow-up period. For the lumefantrine design, patients in the first sampling schedule (Group 1 in Table 1) were assigned to have one sample taken on the first and second days of treatment and three samples taken over the rest of the follow-up, and patients in the second sampling schedule (Group 2 in Table 1) were specified to have one sample taken on the first, second and third days with an additional two samples taken over the follow-up.

**Table 1:** Optimal sampling times (h) and sampling windows for each drug

Drug		Optimal times				
		(sampling windows)				
Mefloquine (8.3 mg/kg at 0, 24 and 48 h)	Group 1:	3.22	43.0	147*	496	1035
	(n = 50)	(2.35, 4.11)	(39.4, 47.6)	(139, 158)	(457, 546)	(988, 1058)
	Group 2:	2.02	28.0	67.8	147*	538
	(n = 50)	(1.49, 2.81)	(26.7, 29.6)	(62.7, 75.8)	(139, 158)	(456, 593)
Mefloquine (15 mg/kg at 24 h, 10 mg/kg at 48 h)	Group 1:	26.5	66.9	147*	544	1011
	(n = 50)	(25.8, 27.8)	(60.8, 71.7)	(134, 158)	(501, 593)	(971, 1058)
	Group 2:	26.5	66.3	66.5	147*	694
	(n = 50)	(25.8, 27.5)	(60.5, 71.6)	(60.5, 71.7)	(141, 158)	(650, 776)
Lumefantrine (12 mg/kg at 0, 8, 24, 36, 48 and 60 h)	Group 1:	2.28	30.3	100	147*	267
	(n = 50)	(1.78, 2.78)	(28.1, 31.7)	(86.9, 110)	(132, 159)	(231, 310)
	Group 2:	11.6	37.7	53.7	147*	218
	(n = 50)	(10.1, 13.4)	(37.7, 41.6)	(52.3, 56.6)	(130, 149)	(203, 261)
Piperaquine (18 mg/kg at 0, 24 and 48 h)	Group 1:	0.18	30.0	77.3	147*	705
	(n = 50)	(0.10, 0.61)	(29.1, 31.9)	(72.3, 84.9)	(138, 156)	(652, 747)
	Group 2:	2.54	24.0	147*	358	1291
	(n = 50)	(2.15, 3.95)	(24.0, 24.6)	(138, 155)	(259, 382)	(1204, 1322)
Desethylamodiaquine (10 mg/kg of amodiaquine at 0, 24 and 48 h)	Group 1:	9.67	24.0	147*	348	651
	(n = 50)	(7.03, 10.1)	(24.0, 24.1)	(129, 157)	(339, 398)	(599, 651)
	Group 2:	0.71	24.0	98.0	147*	348
	(n = 50)	(0.38, 1.36)	(24.0, 24.1)	(88.4, 106)	(129, 157)	(316, 397)

*3^rd^ hour of the seventh day of treatment.

### Evaluation of the optimal designs

Additional file 1: Table S2 reports the expected and empirical %RSEs for the mefloquine optimal design employing a dosing regimen of 8.3 mg/kg daily for 3 days and Additional file 1: Table S3 displays the same information using a dosing regimen of 15 mg/kg on the second day of artesunate/mefloquine combination therapy and 10 mg/kg on the third day. For all study populations, the one-compartment models exhibited acceptable empirical %RSEs for the PK, BSV and residual variance parameters (≲ 25%, ≲ 60%, ≲z 50%, respectively). The only exception was the BSV of *CL*/*F* for children in Additional file 1: Table S3, which was slightly high. When attempting to estimate all parameters of the two-compartment model reported for non-pregnant adults, the empirical %RSEs for the PK parameters were satisfactory but unacceptably high (≥ 100%) for the BSVs of inter-compartmental clearance (*Q*/*F*) and *V*_*p*_/*F*. Therefore the BSV of *Q/F* was fixed to the value it was simulated at. This resulted in acceptable empirical %RSEs in Additional file 1: Table S3 (though slightly high for *Q/F*), and for Additional file 1: Table S2 resulted in a high but reduced empirical %RSE for the BSV of *V*_*p*_/*F* and slightly high %RSEs for *Q/F* and *V*_*p*_/*F*. All other empirical %RSEs in Additional file 1: Table S2 were acceptable.

Additional file 1: Table S4 reports the expected and empirical %RSEs for the lumefantrine optimal designs. The design yielded sufficient information for the estimation of two-compartment model parameters reported for pregnant women. The empirical %RSEs for the one-compartment model reported in children were acceptable with the exception of *k*_*a*_, which was nearly twice as high as the nominal 25%. Due to a structural identifiability problem with the model reported for non-pregnant adults, the absorption rate constant (*k*_*a*_) was declared as fixed in POPT for the non-pregnant adults, as well as the BSVs of *k*_*a*_ and *V*_*p*_/*F* . Consequently, *k*_*a*_ and its BSV were fixed in the estimation step of the evaluation procedure and the BSVs of *Q/F* and *V*_*p*_/*F* were omitted. After these adjustments, the empirical %RSEs for the estimated parameters were satisfactory.

Additional file 1: Table S5 displays the results from the evaluation of the piperaquine design. For the non-pregnant adult model reported in [19], the design yielded acceptable precision for all of the PK parameters with the exception of *k*_*a*_, which displayed an empirical %RSE that was high but less than 50%. The empirical precision for the BSVs of *CL/FQ/F* and *V*_*p*_/*F* was marginally higher than the nominal 60% and for the BSVs of *k*_*a*_ and *V*_*c*_/*F* was acceptable. All parameters of the two-compartment model fitted to the simulated data for non-pregnant women displayed acceptable empirical precision. For the paediatric model reported in [18], the BSVs of *V*_*c*_/*FQ/F* and *V*_*p*_/*F* were declared as fixed in POPT due to unacceptably high expected %RSEs (>100%). These parameters were reported to have additive between-subject variability, which was specified in POPT. However, in the simulation-estimation procedure it was assumed that all PK parameters were lognormally distributed, which resulted in satisfactory empirical precision for all parameters except the BSV of *V*_*c*_/*F* (see Discussion for a full description of the rationale). The two-compartment model fitted to paediatric data simulated from the model reported in [26] displayed acceptable empirical %RSEs for all parameters.

Additional file 1: Table S6 shows the expected and empirical %RSEs for the desethylamodiaquine optimal design. For the children the empirical %RSEs for all reported parameters were acceptable. For the adults, acceptable empirical precision was observed for all parameters of the unpublished two-compartment model (see Additional file 1 for model details).

## Discussion

This study proposes optimal designs that can be used prospectively to study the population pharmacokinetics of the partner drugs used with the most widely used ACT. The designs were based on models from the literature and the key specifications of 100 patients with five samples per person. The sampling schedules for each drug are applicable to the target populations of non-pregnant adults, pregnant women and children. Sampling windows were derived for each optimal sampling time, which provide flexibility for taking samples in the field and capture standard days of follow-up for clinical studies (days 1, 2, 3, 7, 14, 21, 28, 35, 42, 49, 56 and 63).

The design for mefloquine was based primarily on one-compartment models, which in general displayed acceptable expected and empirical precision for all target populations. The only exception was the BSV of *CL*/*F* for children in Additional file 1: Table S3, but this result was due to the conservative approach to evaluating the designs. The designs were derived in POPT assuming 100 patients, which means that the expected %RSEs were calculated assuming 100 patients for any given competing model. However, the simulation-estimation procedure was performed on only 33 or 34 patients. To ensure the design provided adequate information for children receiving two doses of mefloquine, the simulation-estimation procedure was performed again with 50 simulated children and resulted in acceptable empirical %RSEs for all parameters. Empirical precision was not ideal for all parameters of the two-compartment model for non-pregnant adults, but again, this was due to the conservative evaluation procedure. The simulation-estimation procedure was performed again with 65 simulated adult patients and resulted in acceptable empirical %RSEs for all parameters. Therefore the proposed design should be sufficient for estimating a two-compartment model for non-pregnant adults, and as more studies are conducted the designs can be updated to provide more information for estimating two-compartment models in all populations.

The lumefantine design resulted in precise estimation of the two-compartment model parameters for pregnant women. The design yielded acceptable precision for the estimable parameters for the non-pregnant adults, but was not optimized for *k*_*a*_ or the BSVs of *k*_*a*_ or *V*_*p*_/*F* for this group due to a structural identifiability problem with the model reported in [15]. This issue was explored in POPT by increasing the number of samples to eight per patient, but still resulted in unacceptably high expected %RSEs for these parameters. This model was entered into POPT since it was the only published model for non-pregnant adults, however it is likely that other two-compartment profiles exist for this group. Therefore, it is recommended to vary sampling within the windows for non-pregnant adults to investigate other plausible parameter values. For the children, the design provided adequate information for most of the one-compartment model parameters reported in [17], however, the reported model was estimated from concentrations that were taken only up to 72 h, which is well before the distribution phase is complete (approximately 14 days). Thus there may be other plausible structural PK models for children (e.g. a two-compartment model, which is quite likely given the adult profiles), and it is, therefore, recommended to vary sampling within the windows to provide means for exploring alternative structural models for this group. As more population PK studies of lumefantrine are performed, this design will be updated to account for other reported models in all study populations, particularly those in children and non-pregnant adults.

The piperaquine design was based on two-compartment models and exhibited acceptable expected precision of all estimable parameters for all study populations. For the non-pregnant adults, the empirical %RSEs for *k*_*a*_ and the BSVs of *CL*/*FQ/F* and *V*_*p*_*/F* were slightly greater than the target values, but as with the mefloquine design this was due to the conservative approach to evaluation. The simulation-estimation procedure was repeated for this group with 50 patients per simulated dataset and resulted in acceptable %RSEs for all parameters. The two-compartment models fitted to data simulated from models reported in [27] for pregnant and non-pregnant women yielded acceptable empirical precision for all parameters. The simulation models used for these evaluations provided a detailed description of the PK for these groups (three compartment models with n-transit absorption), and under the proposed design allowed the two-compartment models to be fitted with a lag-time (fixed) as well as covariances on the BSVs (*CL*/*FV*_*c*_/*FQ*/*F* and *V*_*p*_/*F*). This empirical result provides evidence that the proposed sampling schedules should be sufficient for estimating two-compartment models in these patient groups. The design was not optimized for the BSVs of *V*_*c*_/*FQ*/*F* and *V*_*p*_/*F* reported in [18] for children since POPT gave unacceptably high expected %RSEs. However, these parameters were reported to have additive between-subject variability, thus these BSVs were declared as additive in POPT. Though it is possible that the between-subject variability for these parameters is truly additive, it is biologically plausible and thus common practice to assume exponential between-subject variability for all PK parameters. Consequently, the simulation-estimation procedure for children was performed assuming exponential between-subject variability, and resulted in acceptable empirical precision for all parameters except the BSV of *V*_*c*_/*F*. Again, this result was due to the conservative approach to evaluating the designs, and when repeated with 50 patients per simulated dataset all parameters displayed acceptable %RSEs. The two-compartment model fitted to the simulated paediatric data from [26] yielded acceptable empirical precision for all parameters. As with the adults, the simulation model used for this evaluation provided a detailed description of the PK for this group, and under the proposed design allowed estimation of covariances between the BSV parameters. Therefore the proposed optimal design should provide reasonable means for estimating two-compartment models in all patient populations, and it is recommended to vary sampling within the windows to gain more knowledge of their PK profiles.

The design for desethylamodiaquine resulted in acceptable expected and empirical precision for children and adults. However, it is important to note that the model reported for adults was estimated from pregnant and post-partum women with *Plasmodium vivax* malaria. Therefore, it is possible that adult patients with *Plasmodium falciparum* malaria may display different PK profiles than those simulated, and men may display different profiles than non-pregnant women. Furthermore, Stepniewska *et al.*[21] only reported a BSV for *CL*/*F*, but BSVs for the other PK parameters may well exist in children. Thus the proposed sampling schedule should be considered a truly “initial” optimal design, and it is, therefore, recommended to vary sampling within the windows to provide flexibility for the exploration of alternative values for the PK and BSV parameters in all patients with uncomplicated *falciparum* malaria. Furthermore, taking additional samples, if possible, may aid this exploration. As more studies of amodiaquine and desethylamodiaquine are performed, the sampling schedules proposed in this paper will be revised to provide designs that take into account newly reported values of PK, BSV and residual error parameters.

## Conclusions

Optimal design methodology allowed the determination of robust blood sampling schedules that utilize current knowledge of the pharmacokinetics of the partner drugs and the practical issues involved with taking blood samples from patients. The proposed designs are economical and efficient, and should be considered when conducting population PK studies of the partner drugs co-administered with the artemisinin derivatives when intensive sampling over the follow-up period is not possible. As with the optimal designs derived for population PK studies of dihydroartemisinin following oral artesunate [10], the designs presented in this paper can be considered a prototype for an iterative open access design support tool to help investigators studying anti-malarial efficacy and pharmacology in field studies. These will be provided by the clinical pharmacology arm of the World Wide Antimalarial Resistance Network (WWARN) [28].

## Competing interests

The authors declare that they have no competing interests.

## Authors’ contributions

JAS and KMJ conceived the project. KMJ, JAS and SBD implemented the designs in POPT. KMJ wrote the first draft of the manuscript. JAS, SBD, JT, NL, and NJW revised the manuscript critically for important intellectual content. All authors read and approved the final manuscript.

## Supplementary Material

Additional file 1 Displays the unpublished two-compartment model for desethylamodiaquine and the results from the evaluation of the designs [29]. Click here for file
