# Supplementary material for: Optimal designs for population pharmacokinetic studies of the partner drugs co-administered with artemisinin derivatives in patients with uncomplicated falciparum malaria
Source: Malar J. 2012 Jul 11;11:143. doi: 10.1186/1475-2875-11-143 (PMC3394219; doi:10.1186/1475-2875-11-143)
Supplement: Additional file 1 — Displays the unpublished two-compartment model for desethylamodiaquine and the results from the evaluation of the designs [29]. [file 1475-2875-11-143-S1.pdf]

## Unpublished model for desethylamodiaquine

JT estimated a two-compartment model with first-order absorption and elimination from rich desethylamodiaquine concentration-time data. For full details of the study from which the data were collected see [28]. Two competing sets of PK parameter values for this model were entered into POPT (Table S1, both sets provided an adequate description of the data). The estimates of the BSVs for $k_a$ and $V_p/F$ were effectively zero ($\leq$0.001%), but to account for larger plausible values that may be encountered in future studies they were set to 50% and 30%, respectively, in POPT. Furthermore, since the estimates of the BSVs for the other parameters were also quite small, they were set to 30% in POPT for the same reason.

The second set of parameter values listed in Table S1 was used for the simulation-estimation procedure. Strong correlation was observed between the BSVs of $CL/F$ and $V_c/F$, so a full variance-covariance matrix was specified for the BSVs of $CL/F$, $V_c/F$ and $Q/F$ in the estimation step.

## Results from the evaluation of the designs

Tables S2-S6 display the expected and empirical %RSEs of model parameters for each optimal design.

Table S1: Parameter estimates for the unpublished two-compartment model for desethylamodiaquine

| | Parameter set 1 | | Parameter set 2$^{\natural}$ | |
|---|---|---|---|---|
| Parameter | Estimate | BSV$^*$ | Estimate | BSV$^*$ |
| $k_a$ (/h) | 0.03 | -$^{\dagger}$ | 0.70 | -$^{\dagger}$ |
| $CL/F$ (L/h) | 32.2 | 0.06 | 32.1 | 0.06 |
| $V_c/F$ (L) | 106 | 0.07 | 2530 | 0.07 |
| $Q/F$ (L/h) | 42.9 | 0.03 | 37.4 | 0.03 |
| $V_p/F$ (L) | 6940 | -$^{\ddagger}$ | 5310 | -$^{\ddagger}$ |
| $\sigma^{\flat}$ | 0.35 | 0$^{\sharp}$ | 0.35 | 0$^{\sharp}$ |

$^*$Between-subject variance

$^{\dagger}$Effectively zero; set to 0.50 in POPT

$^{\ddagger}$Effectively zero; set to 0.30 in POPT

$^{\flat}$Additive on log scale

$^{\sharp}$Fixed to 0

$^{\natural}$Used for the simulation-estimation procedure

Table S2: Expected and empirical percent relative standard errors (%RSEs) of model parameters assuming the optimal design for mefloquine, with a dosing regimen of 8.3 mg/kg at 0, 24 and 48 hours

| | PK parameters | | | | | Between-subject variability | | | | | Residual error | |
|---|---|---|---|---|---|---|---|---|---|---|---|---|
| | $k_a$ | $CL/F$ | $V_c/F$ | $Q/F$ | $V_p/F$ | $k_a$ | $CL/F$ | $V_c/F$ | $Q/F$ | $V_p/F$ | Additive | Proportional |
| **Optimal design** | | | | | | | | | | | | |
| Non-pregnant adults | | | | | | | | | | | | |
| POPT$^{*,\triangle}$ | 9.71 | 3.36 | 4.96 | - | - | 28.4 | 23.9 | 15.1 | - | - | 8.85 | 13.2 |
| Simulation-estimation (n=33)$^{\dagger,\sharp,\S}$ | 7.49 | 5.79 | 8.37 | - | - | - | 36.0 | 27.0 | - | - | 7.14 | - |
| POPT$^{**,\square}$ | 10.9 | 8.00 | 4.64 | 15.1 | 30.2 | 30.7 | 25.6 | 26.1 | 28.1 | 44.8 | 11.8 | 10.1 |
| Simulation-estimation (n=33)$^{\ddagger,\flat,\S}$ | 12.7 | 12.3 | 8.72 | 39.7 | 35.8 | - | 58.2 | 46.1 | - | 72.4 | - | 10.7 |
| Pregnant women | | | | | | | | | | | | |
| POPT$^{*,\diamond}$ | 13.3 | 3.51 | 5.05 | - | - | 48.6 | 23.0 | 15.5 | - | - | 7.19 | 13.0 |
| Simulation-estimation (n=33)$^{\dagger,\natural,\S}$ | 17.9 | 5.15 | 8.21 | - | - | 50.3 | 49.0 | 33.9 | - | - | 9.90 | - |
| Children | | | | | | | | | | | | |
| POPT$^{*,\triangle}$ | 14.7 | 6.07 | 3.59 | - | - | 55.7 | 27.5 | 23.4 | - | - | 7.35 | 22.6 |
| Simulation-estimation (n=34)$^{\dagger,\nabla,\S}$ | 19.9 | 8.25 | 5.54 | - | - | - | 60.5 | 38.6 | - | - | 7.92 | - |

$^{*}$Median expected %RSEs across competing one-compartment models

$^{\triangle}$Proportional residual error not reported, set to 10%; BSV of $k_a$ not reported, set to 50%

$^{\dagger}$Empirical %RSEs for the one-compartment model

$^{\sharp}$BSV of $k_a$ and proportional residual error not reported in [23]

$^{**}$Median expected %RSEs across competing two-compartment models

$^{\square}$BSV of $k_a$ not reported, set to 50%; additive residual error set to 179 ng/mL

$^{\ddagger}$Empirical %RSEs for the two-compartment model

$^{\flat}$BSV of $k_a$ and additive residual error not reported in [14]

$^{\diamond}$BSVs and residual errors set to those for the non-pregnant adults

$^{\natural}$Assumed BSVs and additive residual error as reported in [23]; assumed BSV of $k_a$ of 50%

$^{\nabla}$BSV of $k_a$ and proportional residual error not reported in [12]

$^{\S}$All NONMEM runs successful

Table S3: Expected and empirical percent relative standard errors (%RSEs) of model parameters assuming the optimal design for mefloquine, with a dosing regimen of 15 mg/kg at 24 hours and 10 mg/kg at 48 hours

| | PK parameters | | | | | Between-subject variability | | | | | Residual error | |
|---|---|---|---|---|---|---|---|---|---|---|---|---|
| | $k_a$ | $CL/F$ | $V_c/F$ | $Q/F$ | $V_p/F$ | $k_a$ | $CL/F$ | $V_c/F$ | $Q/F$ | $V_p/F$ | Additive | Proportional |
| **Optimal design** | | | | | | | | | | | | |
| Non-pregnant adults | | | | | | | | | | | | |
| POPT$^{*,\triangle}$ | 5.84 | 2.28 | 3.46 | - | - | 14.2 | 15.7 | 10.4 | - | - | 7.06 | 7.15 |
| Simulation-estimation (n=33)$^{\dagger,\sharp,\S}$ | 5.36 | 5.54 | 8.90 | - | - | - | 41.6 | 27.5 | - | - | 7.07 | - |
| POPT$^{**,\square}$ | 6.56 | 5.07 | 2.95 | 10.5 | 17.0 | 16.3 | 17.5 | 16.3 | 19.4 | 35.7 | 9.01 | 5.72 |
| Simulation-estimation (n=33)$^{\ddagger,\flat,\S}$ | 12.4 | 11.8 | 10.0 | 33.8 | 25.3 | - | 49.2 | 42.9 | - | 62.1 | - | 10.5 |
| Pregnant women | | | | | | | | | | | | |
| POPT$^{*,\diamond}$ | 7.35 | 2.49 | 3.50 | - | - | 22.9 | 16.2 | 10.6 | - | - | 5.63 | 6.68 |
| Simulation-estimation (n=33)$^{\dagger,\natural,\S}$ | 16.2 | 6.03 | 9.17 | - | - | 38.2 | 51.8 | 28.3 | - | - | 8.61 | - |
| Children | | | | | | | | | | | | |
| POPT$^{*,\triangle}$ | 7.66 | 4.13 | 2.24 | - | - | 23.3 | 18.5 | 13.1 | - | - | 5.86 | 11.7 |
| Simulation-estimation (n=34)$^{\dagger,\nabla,\S}$ | 11.2 | 8.90 | 4.81 | - | - | - | 73.4 | 37.3 | - | - | 7.34 | - |

$^{*}$Median expected %RSEs across competing one-compartment models
$^{\triangle}$Proportional residual error not reported, set to 10%; BSV of $k_a$ not reported, set to 50%
$^{\dagger}$Empirical %RSEs for the one-compartment model
$^{\sharp}$BSV of $k_a$ and proportional residual error not reported in [23]
$^{**}$Median expected %RSEs across competing two-compartment models
$^{\square}$BSV of $k_a$ not reported, set to 50%; additive residual error set to 179 ng/mL
$^{\ddagger}$Empirical %RSEs for the two-compartment model
$^{\flat}$BSV of $k_a$ and additive residual error not reported in [14]
$^{\diamond}$BSVs and residual errors set to those for the non-pregnant adults
$^{\natural}$Assumed BSVs and additive residual error as reported in [23]; assumed BSV of $k_a$ of 50%
$^{\nabla}$BSV of $k_a$ and proportional residual error not reported in [12]
$^{\S}$All NONMEM runs successful

Table S4: Percent relative standard errors (%RSEs) of model parameters assuming the optimal design for lumefantrine, with a dosing regimen of 12 mg/kg at 0, 8, 24, 36, 48 and 60 hours

| | PK parameters | | | | | Between-subject variability | | | | | Residual error | |
|---|---|---|---|---|---|---|---|---|---|---|---|---|
| | $k_a$ | $CL/F$ | $V_c/F$ | $Q/F$ | $V_p/F$ | $k_a$ | $CL/F$ | $V_c/F$ | $Q/F$ | $V_p/F$ | Additive | Proportional |
| **Optimal design** | | | | | | | | | | | | |
| Non-pregnant adults | | | | | | | | | | | | |
| POPT$^{*,\triangle,\ddagger}$ | - | 5.53 | 17.7 | 11.6 | 21.9 | - | 18.9 | 18.0 | 50.3 | - | 5.92 | 16.8 |
| Simulation-estimation (n=33)$^{\dagger,\sharp,\S}$ | - | 10.7 | 23.7 | 17.6 | 18.5 | - | 40.7 | 34.4 | - | - | 9.07 | - |
| Pregnant women | | | | | | | | | | | | |
| POPT$^{*,\natural}$ | 5.32 | 5.28 | 15.9 | 8.50 | 8.36 | 24.5 | 14.4 | 16.0 | 16.2 | 15.9 | - | 10.3 |
| Simulation-estimation (n=33)$^{\dagger,\S}$ | 10.3 | 5.77 | 21.4 | 10.0 | 10.7 | 34.9 | 29.6 | 41.0 | 33.6 | 28.6 | - | 19.3 |
| Children | | | | | | | | | | | | |
| POPT$^{*,\natural,\nabla}$ | 22.2 | 5.97 | 8.99 | - | - | 24.3 | 16.8 | 16.6 | - | - | - | 4.58 |
| Simulation-estimation (n=34)$^{\dagger,\flat,\S}$ | 45.2 | 6.01 | 15.7 | - | - | 41.7 | - | 34.7 | - | - | - | 9.49 |

$^{*}$Median expected %RSEs across competing models
$^{\triangle}$Proportional residual error not reported; set to 10%
$^{\ddagger}$ $k_a$ and the BSVs of $k_a$ and $V_p/F$ were fixed due to a structural identifiability problem with the model reported in [15]
$^{\dagger}$Empirical %RSEs
$^{\sharp}$ $k_a$ and its BSV were fixed to the values reported in [15] and the BSVs of $Q/F$ and $V_p/F$ were omitted from the estimation
$^{\natural}$Additive residual error set to 23 ng/mL and declared as fixed
$^{\nabla}$BSV of $CL/F$ not reported in [17], set to 30%
$^{\flat}$ BSV of $CL/F$ not reported in [17]; additive residual error was fixed to the value reported in [17]
$^{\S}$No. of successful NONMEM runs: non-pregnant adults, 99; pregnant women, 100; Children, 97

Table S5: Percent relative standard errors (%RSEs) of model parameters assuming the optimal design for piperaquine, with a dosing regimen of 18 mg/kg at 0, 24 and 48 hours

| | PK parameters | | | | | Between-subject variability | | | | | Residual error | |
|---|---|---|---|---|---|---|---|---|---|---|---|---|
| | $k_a$ | $CL/F$ | $V_c/F$ | $Q/F$ | $V_p/F$ | $k_a$ | $CL/F$ | $V_c/F$ | $Q/F$ | $V_p/F$ | Additive | Proportional |
| **Optimal design** | | | | | | | | | | | | |
| Non-pregnant adults | | | | | | | | | | | | |
| POPT$^{*,\triangle}$ | 20.2 | 4.69 | 11.2 | 11.1 | 7.56 | 18.9 | 17.2 | 16.8 | 25.5 | 30.9 | - | 8.65 |
| Simulation-estimation (n=33)$^{\dagger,\S}$ | 46.1 | 10.0 | 22.7 | 18.7 | 11.2 | 51.5 | 66.3 | 33.8 | 62.7 | 69.4 | - | 16.0 |
| Simulation-estimation (n=33)$^{\dagger,\ddagger,\S}$ | 24.3 | 8.71 | 26.5 | 18.9 | 9.18 | 63.0 | 33.1 | 51.3 | 43.4 | 42.9 | - | 11.9 |
| Pregnant women | | | | | | | | | | | | |
| POPT$^{*,\triangle}$ | 20.1 | 4.61 | 11.2 | 11.7 | 7.50 | 19.5 | 17.0 | 16.6 | 29.2 | 31.1 | - | 9.40 |
| Simulation-estimation (n=33)$^{\dagger,\diamond,\S}$ | 19.7 | 9.22 | 21.7 | 15.6 | 8.69 | 49.8 | 50.0 | 58.6 | 53.7 | 57.3 | - | 12.0 |
| Children | | | | | | | | | | | | |
| POPT$^{*,\triangle,\sharp}$ | 9.68 | 3.43 | 11.6 | 4.79 | 2.86 | 21.3 | 18.7 | - | - | - | - | 3.85 |
| Simulation-estimation (n=34)$^{\dagger,\flat,\natural,\S}$ | 19.2 | 3.39 | 33.6 | 13.9 | 13.1 | 63.5 | - | 122.6 | 56.7 | 48.2 | - | 10.3 |
| Simulation-estimation (n=34)$^{\dagger,\nabla,\S}$ | 19.0 | 8.52 | 10.8 | 8.88 | 8.47 | 50.2 | 36.8 | 35.8 | 39.9 | 46.4 | - | 14.4 |

$^{*}$Median expected %RSEs across competing models

$^{\triangle}$Additive residual error not reported in [18, 19]; set to 1 ng/mL and declared as fixed

$^{\dagger}$Empirical %RSEs

$^{\ddagger}$Data simulated from [27]; analyzed with a two-compartment model (with fixed lag-time)

$^{\diamond}$Data simulated from [27]; analyzed with a two-compartment model

$^{\sharp}$BSVs of $V_c/F$, $Q/F$ and $V_p/F$ set as additive in POPT and declared fixed; BSV of $CL/F$ not reported, set to 30% (additive) in POPT

$^{\flat}$BSV of $CL/F$ not reported in [18]

$^{\natural}$Between-subject variability assumed exponential

$^{\nabla}$Data simulated from [26]; analyzed with a two-compartment model (with fixed lag-time)

$^{\S}$No. of successful NONMEM runs: non-pregnant adults, 100; pregnant women, 100; Children, 85 for data simulated from [18], 88 for data simulated from [26]

Table S6: Percent relative standard errors (%RSEs) of model parameters assuming the optimal design desethylamodiaqine, with a dosing regimen of 10 mg/kg of amodiaquine at 0, 24 and 48 hours

| | PK parameters | | | | | Between-subject variability | | | | | Residual error | |
|---|---|---|---|---|---|---|---|---|---|---|---|---|
| | $k_a$ | $CL/F$ | $V_c/F$ | $Q/F$ | $V_p/F$ | $k_a$ | $CL/F$ | $V_c/F$ | $Q/F$ | $V_p/F$ | Additive | Proportional |
| **Optimal design** | | | | | | | | | | | | |
| Adults | | | | | | | | | | | | |
| POPT$^{*,\triangle}$ | 14.0 | 4.56 | 11.0 | 9.57 | 8.15 | 31.9 | 18.1 | 61.8 | 38.4 | 29.3 | 13.7 | 8.21 |
| Simulation-estimation (n=66)$^{\dagger,\sharp,\S}$ | 13.5 | 3.94 | 7.45 | 9.59 | 7.62 | - | 29.9 | 49.3 | 64.3 | - | - | 5.71 |
| Children | | | | | | | | | | | | |
| POPT$^{*,\triangle,\flat}$ | 19.2 | 4.43 | 15.9 | 11.0 | 8.24 | 48.2 | 29.2 | 31.7 | 66.6 | 33.1 | 12.3 | 6.78 |
| Simulation-estimation (n=34)$^{\dagger,\natural,\S}$ | 21.7 | 5.55 | 17.9 | 19.0 | 11.9 | - | 44.8 | - | - | - | - | 7.32 |

$^*$Median expected %RSEs across competing models

$^\triangle$Additive component of residual error not reported in [21] or estimated in the model provided by JT; assumed to be 10 ng/mL in POPT

$^\dagger$Empirical %RSEs

$^\sharp$Simulation-estimation performed on non-pregnant adults and pregnant women together (with allometric scalling on clearance and volume parameters)

$^\flat$Only the BSV for $CL/F$ was reported in [21]; BSV for $k_a$ set to 50% and BSVs for other parameters set to 30%

$^\natural$Only the BSV for $CL/F$ was reported in [21]

$^\S$All NONMEM runs successful
